# EPIC: an effective low toxicity regimen for relapsing lymphoma.

**DOI:** 10.1038/bjc.1993.393

**Published:** 1993-09

**Authors:** T. Hickish, A. Roldan, D. Cunningham, J. Mansi, S. Ashley, V. Nicolson, M. E. Gore, D. Catovsky, I. E. Smith

**Affiliations:** Lymphoma Unit, Royal Marsden Hospital, Sutton, Surrey, London, UK.

## Abstract

We have treated 40 patients was relapsed or resistant lymphoma with the combination of Etoposide, Prednisolone, Ifosfamide and Cisplatin (EPIC). Complete response was obtained in 11 patients (28%) with an overall response of 58%. The presence of bulky disease (P < 0.005), elevated LDH serum levels (P < 0.005), response to prior chemotherapy (P < 0.01) and B symptoms (P < 0.005) were significantly associated with response. However on multivariate analysis only the presence of bulky disease and of B symptoms were independent adverse factors for response and for survival. The regimen was well tolerated with myelosuppression being the most common toxicity. Leucopenia < or 1,000 microliters-1 and thrombocytopenia < or = 25,000 microliters-1 developed in 27% and 4% of cycles respectively. There were no treatment related deaths. The EPIC regimen has equivalent activity to other reported cisplatin based regimens used in the treatment of recurrent lymphoma, but is associated with lower treatment related morbidity and mortality.


 © Macmillan Press Ltd., 1993

### EPIC: an effective low toxicity regimen for relapsing lymphoma

T. Hickish[1,4], A. Roldan[1], D. Cunningham[1,4], J. Mansi[1], S. Ashley[3], V. Nicolson[2], M.E. Gore[1], D. Catovsky[1] & I.E. Smith[1]

[1]*Lymphoma Unit, Royal Marsden Hospital, Downs Road, Sutton, Surrey SM2 5PT and Fulham Road, London SW3 6JJ;* [2]*Department of Radiology and* [3]*Department of Computing, Royal Marsden Hospital, Downs Road, Sutton, Surrey SM2 5PT;* [4]*CRC Section of Medicine, Institute of Cancer Research, Royal Marsden Hospital, Sutton, Surrey, SM2 5PT, UK.*

**Summary** We have treated 40 patients was relapsed or resistant lymphoma with the combination of Etoposide, Prednisolone, Ifosfamide and Cisplatin (EPIC). Complete response was obtained in 11 patients (28%) with an overall response of 58%. The presence of bulky disease ($P<0.005$), elevated LDH serum levels ($P<0.005$), response to prior chemotherapy ($P<0.01$) and B symptoms ($P<0.05$) were significantly associated with response. However on multivariate analysis only the presence of bulky disease and of B symptoms were independent adverse factors for response and for survival. The regimen was well tolerated with myelosuppression being the most common toxicity. Leucopenia $\leqslant 1,000\,\mu l^{-1}$ and thrombocytopenia $\leqslant 25,000\,\mu l^{-1}$ developed in 27% and 4% of cycles respectively. There were no treatment related deaths. The EPIC regimen has equivalent activity to other reported cisplatin based regimens used in the treatment of recurrent lymphoma, but is associated with lower treatment related morbidity and mortality.

Most patients with aggressive non-Hodgkin's lymphoma (NHL) and Hodgkin's disease (HD) relapsing or resistant to front line chemotherapy have a poor prognosis (De Vita *et al.*, 1989; Cabanillas *et al.*, 1990; Longo, 1990). Although good initial responses have been reported with several salvage regimens, toxicity is significant and long term disease free survival low (Cabanillas *et al.*, 1982; Cabanillas *et al.*, 1987; Velasquez *et al.*, 1988; Hagemeister *et al.*, 1987; Santoro *et al.*, 1986). There is therefore a requirement for more effective low toxicity regimens. In an attempt to meet this need we devised a new salvage chemotherapy combination which includes etoposide, prednisolone, ifosfamide and cisplatin (EPIC). These drugs have different mechanisms of action (Achterrath *et al.*, 1982; Colvin, 1982; Zwelling & Kohn, 1979; Plooy *et al.*, 1984). The biological rationale for this schedule is derived from the single agent activity of these drugs in lymphoma, (Cavalli *et al.*, 1981; Rodriguez *et al.*, 1978; Taylor *et al.*, 1982) the *in vitro* and *in vivo* data suggesting synergy between them (Achterrath *et al.*, 1982; Schabel *et al.*, 1979; Dewinko *et al.*, 1976; Frei *et al.*, 1988; Goldin, 1982; Durand & Goldie, 1987) and the lack of cross resistance between cisplatin and drugs used in first line combinations (Schabel *et al.*, 1979). Also there is evidence of incomplete cross resistance between ifosfamide and cyclophosphamide (Hilgard *et al.*, 1983).

Clinical benefits have been reported with the use of these drugs in different schedules (Judson & Wiltshaw, 1985; Scheulen *et al.*, 1983). Furthermore, investigators at the MD Anderson Hospital have demonstrated that in relapsed and resistant lymphoma the substitution of etoposide for vincristine in a combination which also included ifosfamide and methotrexate increased the CR rate from 17% to 37% (Cabanillas *et al.*, 1982; Cabanillas *et al.*, 1980). Similarly the addition of $100\,mg\,m^{-2}$ cisplatin to the ESA schedule (etoposide, Methylprednisolone, cytarabine) raised the overall response rate from 38% to 69% (Cabanillas *et al.*, 1988). Therefore the clinical evidence supports the experimental data indicating activity and at least an additive effect of these drugs in relapsed and resistant lymphoma.

In this study we describe our experience with the EPIC protocol in the management of these patients.

Correspondence: D. Cunningham, Head Lymphoma Unit, Department of Medicine, Royal Marsden Hospital, Sutton, Surrey SM2 5PT, UK.
Received 24 November 1992; and in revised form 5 May 1993.

### Patients and methods

### *Patient selection*

Thirty-two patients with NHL and 10 with HD were enrolled into this trial between November 1989 and March 1991. Eligibility criteria included the following: (1) biopsy proven relapsing or resistant NHL or HD; (2) measurable disease; (3) informed consent; (4) EDTA $\geqslant 60\,ml\,min^{-1}$. Response to prior chemotherapy was defined according to WHO criteria (Miller *et al.*, 1981). Patients were then classified as follows:

- Relapse from prior remission (CR or PR).
- Primary resistant disease; failure to achieve a remission (PR or CR) with any chemotherapy used in the past.

The purpose of this classification was to group patients in terms of the chemosensitivity prior to implementing EPIC chemotherapy.

All but two patients with NHL had been exposed to alkylating agents and anthracyclines in previous combinations. The two exceptions were patients with follicular NHL treated with CVP and one of these had also received high dose therapy plus autologous bone marrow transplantation (ABMT). The patients with HD had all been treated with both a MOPP type and an anthracycline containing combination. In addition, seven had been treated with extended field radiotherapy and three with high dose chemotherapy and bone marrow transplant in the past.

Bulky disease was considered to be present if any mass measured > 5 cm in diameter on CT evaluation or clinical examination.

Patient's characteristics are shown in Table I. Staging was conducted prior to the first cycle of chemotherapy and included clinical examination, full blood count, usual serum chemistries, chest radiograph and computer tomography (CT) scan of chest, abdomen and pelvis. An EDTA clearance test was performed before every other cycle unless clinically indicated. Restaging with the appropriate imaging technique was performed every two courses. MRI and high dose Gallium scans were performed at the completion of therapy if a residual mass was shown on CT.

### *Chemotherapy*

The dose schedule of the EPIC regimen is as follows: Etoposide $100\,mg\,m^{-2}$ intravenous in 500 ml N-saline over 1 h on Days 1–4; Ifosfamide $1\,g\,m^{-2}$ by bolus intavenous on Days 1–5 with hydration and Mesna; Prednisolone 100 mg daily orally on Days 1–5; cisplatin $60\,mg\,m^{-2}$ by short intravenous infusion with hydration and anti-emetics on Day

Table I Patient characteristics

| | No. | % |
|---|---|---|
| All patients | 40 | 100 |
| *Age* Mean (range) | 50 (19–68) | |
| *Gender* | | |
| Male | 23 | 58 |
| Female | 17 | 42 |
| *Histology* | | |
| Intermediate grade NHL | 29 | 73 |
| Diffuse immunoblastic (3 transformed) | 14 | |
| Diffuse large cell (2 transformed) | 7 | |
| Diffuse mixed (1 transformed) | 5 | |
| Follicular large cell | 2 | |
| Peripheral T cell | 1 | |
| Low grade NHL | 2 | 5 |
| Follicular small cleaved | 1 | |
| Follicular mixed | 1 | |
| Hodgkin's disease | 9 | 22 |
| Nodular sclerosis | 7 | |
| Mixed cellularity | 2 | |
| *Number of previous treatments* | | |
| One | 17 | 42 |
| ⩾2 | 23 | 58 |
| *Response to prior therapy* | | |
| CR | 9 | 22 |
| PR | 10 | 25 |
| Primary resistant disease | 21 | 53 |
| *Interval in remission* (19 patients) | | |
| ⩽3 months | 9 | 47 |
| 3–6 months | 6 | 32 |
| 6–12 months | 2 | 11 |
| >12 months | 2 | 11 |
| *Stage* | | |
| IIA | 3 | 8 |
| IIB | 2 | 5 |
| IIIA | 1 | 2 |
| IIIB | 4 | 10 |
| IVA | 11 | 28 |
| IVB | 19 | 47 |
| *BM involvement* | | |
| Yes | 12 | 30 |
| No | 27 | 67 |
| Not investigated | 1 | 3 |
| *Extranodal involvement* | | |
| None | 10 | 25 |
| One site | 13 | 33 |
| ⩾2 sites | 17 | 42 |
| *Bulky disease* | | |
| Present | 19 | 48 |
| Absent | 21 | 52 |
| *B symptoms* | | |
| Present | 25 | 62 |
| Absent | 15 | 38 |
| *LDH serum levels* (25 patients) | | |
| ⩽240 | 14 | 56 |
| >240 (elevated) | 11 | 44 |

10 provided EDTA clearance ⩾ 60 ml $min^{-1}$. Treatment was given as an in-patient and was repeated every 3 weeks.

Chemotherapy was delayed if white cell count < 2,000 $\mu l^{-1}$ or platelets < 100,000 $\mu l^{-1}$ on day one. Cimetidine, co-trimoxazole and antifungal prophylaxis with oral nystatin and amphotericin were given throughout the treatment. The response to EPIC and toxicity were determined using the WHO criteria (Miller *et al.*, 1981).

### *Statistical analysis*

Time to treatment failure (TTF; time to relapse, progression or death) and duration of survival were calculated from the beginning of treatment. Survival curves were estimated by the method of Kaplan and Meier (Peto *et al.*, 1977). The Log rank test was utilised to compared differences in survival and TTF (Peto *et al.*, 1977). The proportional hazards model was used to determine the independence of factors for survival (Cox, 1972). Prognostic factors for response were compared using chi-square, Fisher exact or Mann Whitney non parametric test as indicated, and their independent effect was tested using the logistic regression model (Lehmann, 1959).

### Results

### *Response rates*

Forty patients were evaluable for response. CR was obtained in 11 patients (28%) and PR in 12 (30%), with an overall response rate of 58%. Two patients were excluded from the final analysis of response – one was found to have a second neoplasm instead of a relapsed NHL. The other, a patient with HD who had achieved CR, was excluded because in retrospect we could not exclude an effect of the prior chemotherapy in the response. However these two patients were included in the toxicity analysis. The response rates associated with several prognostic factors are shown in Table II. Patients relapsing from a CR achieved a response of 89%, while only three patients (27%) with primary resistant disease responsed ($P < 0.01$). The overall response rate for NHL 16/31 (48%) was not significantly different from that of HD 7/9 (78%). Stage, bone marrow involvement or number of extranodal sites affected did not correlate with the quality of response and response rate. The presence of B symptoms ($P < 0.05$), bulky disease ($P < 0.005$) and elevated LDH ($P < 0.005$) were poor prognostic features. However on multivariate analysis only bulky disease and the presence of B symptoms were independent predictors of response and survival. Patients with absence of bulky disease and B symptoms (nine patients) had a response rate of 100% compared with 56% of those with only one or them and 8% of those with both bulky disease and B symptoms.

Patients with transformed NHL did worse than other intermediate grade NHL, but the difference was not significant.

Seven patients with HD (78%) had a PR. There were no CRs. Five of these patients were subsequently treated with high dose therapy and marrow transplantation. The other two had already been treated with bone marrow transplantation before EPIC.

### *Time to treatment failure and survival*

TTF and survival are shown in Figures 1, 2 and 3. Median TTF is 18 months for CR and 6 months for PR ($P < 0.005$). Seven (30%) of the patients who responded remain free of disease, with a median follow up of 12 (4–15) months. The median survival for the whole group of patients is 9 months. There is no difference in survival for NHL *vs* HD. The TTF for patients with NHL is marginally better than that for those with HD ($P = 0.07$). Median survival for CR, PR and non responders is 21, 12 and 6 months respectively ($P < 0.005$). Fourteen patients remain alive, with a median follow up of 13 (4–21) months. One died from a second neoplasm soon after achieving PR.

### *Toxicity and chemotherapy*

The EPIC regimen was generally well tolerated. A total of 150 courses of treatment were given to 42 patients, with a median 3 (1–8) cycles. The most frequent significant side-effect was myelosuppression (see Table III). Forty episodes of WHO grade 4 leucopenia (WBC ⩽ 1000 $\mu l^{-1}$) were found in 23 patients. Delay of therapy usually due to myelosuppression or infection, occurred in 20 patients with a median delay of 2 weeks per patient. Twelve episodes of fever with neutropenia (8%) were recorded, including three that were severe (WHO grade 3). Two non-disseminated Herpes-Zoster infections were also found. Alopecia was almost universal. Nausea and vomiting were usually moderate, only one patient developing WHO grade 3 toxicity. Mucositis was rare and haemorrhagic cystitis was not found in our patients. Two patients had reversible impairment of renal function and cisplatin was omitted in one and two courses respectively. The dose of etoposide was reduced by 25% in two patients

**Table II** Response to EPIC regimen

| Patient characteristics | CR No. | CR % | CR + PR No. | CR + PR % | P value (overall response) |
|---|---|---|---|---|---|
| All patients (40) | 11 | 28 | 23 | 58 | |
| *Histology* | | | | | |
| Intermediate grade NHL | 11 | 38 | 14 | 48 | NS |
| Low grade NHL | – | – | 2 | 100 | |
| Hodgkin's disease | – | – | 7 | 78 | |
| *Response to prior chemotherapy* | | | | | |
| CR | 5 | 56 | 8 | 89 | $P<0.05$ |
| PR | 4 | 40 | 6 | 60 | |
| Primary resistant disease | 2 | 10 | 9 | 43 | |
| *B symptoms* | | | | | |
| Present | 5 | 20 | 11 | 44 | $P<0.05$ |
| Absent | 6 | 40 | 12 | 80 | |
| *Bulky disease* | | | | | |
| Present | 4 | 21 | 4 | 21 | $P<0.005$ |
| Absent | 7 | 33 | 19 | 90 | |
| *LDH* | | | | | |
| <240 | 5 | 36 | 11 | 79 | $P<0.005$ |
| >240 (elevated) | 2 | 29 | 2 | 29 | |
| *Extranodal site* | | | | | |
| None | 3 | 30 | 6 | 60 | NS |
| One | 4 | 31 | 8 | 62 | |
| Two or more | 4 | 24 | 9 | 53 | |

NS = non significant.

**Figure 1** Time to treatment failure for patients who achieved either CR (——) or PR (- - -).

**Figure 2** Kaplan and Meier survival curve for patients achieving CR (———), PR (- - -) and non-responders (— — —).

**Figure 3** Time to treatment failure for Hodgkin's disease (———) and non-Hodgkin's lymphoma (- - -).

**Table III** Toxicity

| *Toxicity* | *No. of patients* | *%* |
|---|---|---|
| All patients | 42 | |
| Myelotoxicity (WHO grade 4) | | |
| WBC $<1,000\,\mu l^{-1}$ | 23 | 55 |
| Platelets $<25,000\,\mu l^{-1}$ | 3 | 7 |
| Neutropenic fever | 10 | 24 |
| Grade 3 | 3 | |
| Grade 2 | 7 | |
| Other infections | | |
| Herpes zoster | 2 | 5 |
| Hickman line infection | 1 | 2 |
| Nausea and vomiting | 20 | 48 |
| Grade 3 | 1 | |
| Grade 2 | 7 | |
| Grade 1 | 12 | |
| Mucositis | 2 | 5 |
| Peripheral neuropathy | 2 | 5 |
| Renal toxicity (reversible) | 2 | 5 |
| Ifosfamide encephalopathy (reversible) | 1 | 2 |

following a septic episode. Ifosfamide was reduced by 50% in a patient with tremor. Two other patients had a 20% reduction of ifosfamide, one because of renal impairment and the other following an episode of neutropenic fever. In one patient a dose of cisplatin was omitted because of neutropenia.

In five patients treatment was discontinued after one course of chemotherapy. One had had high dose chemotherapy with ABMT and developed prolonged thrombocytopenia. The other four had progressive disease.

### Discussion

Several therapeutic alternatives have been developed for patients with NHL who had failed first line doxorubicin and cyclophosphamide containing regimens. These include the use of drug combinations, theoretically non cross resistant with first line regimens, the reversal of multidrug resistance and the use of high dose chemotherapy with ABMT.

The role for intensive chemotherapy with ABMT in relapsed NHL is undecided. However Philip *et al.* have reported an actuarial 3 year disease-free survival after ABMT of 0% and 14% for NHL patient with refractory and resistant relapsed disease respectively (Philip *et al.*, 1987). Moreover, long term disease free survival is around 20% in non selected groups of patient, (Takvorian *et al.*, 1987; Appelbaum *et al.*, 1987; Phillips *et al.*, 1990) results not much better than those achieved with conventional salvage therapy alone. This indicates that in relapsed NHL, intensive chemotherapy with ABMT only has a place in patients who have sensitive disease and low tumour burden after salvage therapy (Philip *et al.*, 1987; Takvorian *et al.*, 1987). In patients achieving CR with salvage chemotherapy the advantage of intensive chemotherapy is not clear and the results of ongoing trials, such as the Parma study, are eagerly awaited (Philip *et al.*, 1991). Patients achieving only a PR with a second line chemotherapy have a very poor prognosis, and should probably be offered intensive therapy with ABMT in an attempt to achieve long term remissions.

Another approach is to overcome drug resistance by infusional therapy with doxorubicin and vincristine or by the addition of a P-170 glycoprotein blocking agent to those combinations (Chabner & Wilson, 1991). Miller *et al.* have reported a response rate of 72% in 18 NHL and HD patients using a prolonged continuous infusion of verapamil plus doxorubicin and vincristine together with cyclophosphamide and dexamethasone (Miller *et al.*, 1991).

The EPIC regimen is an attempt to develop a new chemotherapy combination for relapsed and resistant lymphoma non cross resistant with first line regimens. The results of several such combinations have been published (see Table IV). Of particular interest are the series of trials by the MD Anderson Hospital Group. When comparing these regimens in terms of response it is clearly crucial to be mindful of the difference in case selection (Press *et al.*, 1991). Patients with primary resistant lymphomas and resistant relapse do particularly badly (Cabanillas *et al.*, 1982; Cabanillas *et al.*, 1987; Philip *et al.*, 1987; Takvorian *et al.*, 1987; Appelbaum *et al.*, 1987; Phillips *et al.*, 1990). Several othe prognostic features for relapsed lymphoma have been reported. These include the duration of response to first line chemotherapy (Cabanillas *et al.*, 1982) elevated serum LDH, (Cabanillas *et al.*, 1987; Velasquez *et al.*, 1988; Press *et al.*, 1991) presence of bulky disease (Cabanillas *et al.*, 1987), number of sites of disease (Cabanillas *et al.*, 1987) and tumour burden (Velasquez *et al.*, 1988). For HD the duration of initial remission, presence of extranodal disease, LDH level, haemoglobin and number of prior relapses influence the outcome in patients treated with first line chemotherapy (Hagemeister *et al.*, 1987). In the IMVP-16 (ifosfamide, methotrexate and VP-16) trial a response rate of 62% with an impressive CR of 37% was obtained in 52 patients (Cabanillas *et al.*, 1982). However their groups of patients had better prognostic factors that the patients in this study. For example, the CR to prior chemotherapy was 40% *vs* 23% in our group and the duration of response to that therapy was greater than 6 months in 60% of their patients, but in only 14% of ours. The addition of methyl GAG (the MIME protocol) did not improve the response but increased the toxicity (Cabanillas *et al.*, 1987). With the DHAP regimen, a combination of dexamethasone, high dose Ara-C and cisplatin, the MD Anderson group achieved an overall response rate of 57.7% with a CR rate of 31% (Velasquez *et al.*, 1988). Unfortunately, toxicity was severe with a toxic death rate of 17%. This group of 90 patients also had slightly better prognostic features than our group; 48% had achieved a CR with previous chemotherapy. It is of interest that all CR but one were observed in patients with low tumour burden. The results obtained with the DHAP regimen have been confirmed by others (Philip *et al.*, 1991; Press *et al.*, 1991). Goss *et al.* with the DICE regimen (dexamethasone, ifosfamide, cisplatin, etoposide) which is similar to EPIC, achieved a CR in 27% of their patients, but with greater toxicity (Goss *et al.*, 1991).

While seven of nine (78%) patients with HD had a PR, none achieved a CR. The analysis is confounded by the subsequent use of high dose chemotherapy and ABMT in three of the seven responding patients before a maximum response was achieved. As previously stated, a third of the patients had prior high dose chemotherapy and ABMT. In one of them EPIC had to be stopped after one course due to marrow failure, the other two attained a PR. Other investigators have obtained CR in 13%–44% of patients relapsing after MOPP and ABVD type combinations (Hagemeister *et al.*, 1987; Santoro *et al.*, 1986; Pfeundschuh *et al.*, 1987; Tseng *et al.*, 1987). Of particular interest are the results of the Italian (Santoro *et al.*, 1986) and German (Pfreundschuh

**Table IV** Salvage therapy in relapsed/refractory lymphoma

| Regimen | Ref. | No. of pats. | CR with prior Tx. | % Patients Response (CR) | Toxic deaths | Granulocytopenic fever | Granulocytopenia $\leqslant 500\,\mu l^{-1}$ | Median survival (months) | Median TTF (CR) (months) |
|---|---|---|---|---|---|---|---|---|---|
| IMVP-16 (MD Anderson) | Cabanillas 1982 | 52 | 40 | 62 (37) | 4 | nm | nm | 15[a] | 12[b] |
| MIME-NHL (MD Anderson) | Cabanillas 1987 | 208 | 42 | 60 (24) | 6 | 59 | nm | 9 | 15[c] |
| IMVP-16/MIME (Amsterdam) | Huijgens | 18 | 33 | 50 (11) | 6 | nm | 95 | BMT | BMT |
| DHAP (MD Anderson) | Velasquez | 90 | 42 | 57.5 (31) | 17 | 48 | 53[d] | 6 | 15 |
| VIP (Indianapolis) | Nichols | 28 | 29 | 36 (8) | 4 | 44 | nm | 7 | nm |
| DICA (Ontario) | Goss | 22 | 41 | 77 (27) | 9 | 41 | 41 | nm | nm |
| DHAP (Wash'ton) | Press | 39 | nm | 67 (23) | 1 | 44 | 74 | BMT | BMT |
| EPIC (R.M.H.) | | 40 | 23 | 58 (28) | 0 | 24 | 55[e] | 9 | 18 |

nm = non mentioned [a]Intermed. gr NHL; [b]All responders, measured from onset of response; [c]Only responders; [d]Granulocytopenia $<300\,\mu l^{-1}$; [e]WBC $\leqslant$ 1000. BMT = Bone marrow transplant.

*et al.*, 1987) groups, who achieved good CR (40 and 44% respectively) with low toxicity in patients with poor prognostic features. Published follow up is short, however. Results of intensive therapy in these patients are promising (Vose *et al.*, 1990).

The EPIC regimen was associated with manageable toxicity in our group of heavily pretreated patients. There were no toxic deaths and the low incidence of febrile episodes associated with neutropenia in our group (8%) compares favourably with the other regimens (Cabanillas *et al.*, 1982, Cabanillas *et al.*, 1987; Velasquez *et al.*, 1988; Hagemeister, 1987; Phillips *et al.*, 1990; Press *et al.*, 1991; Goss *et al.*, 1991; Huijgens *et al.*, 1988; Nichols *et al.*, 1988). Prophylactic cotrimoxazole may have contributed to this low infection rate and absence of mortality.

The poor outcome in the transformed group of lymphomas has been previously found by some (Armitage *et al.*, 1981) but not by other authors (Acker *et al.*, 1983).

Dose intensity is an accepted aim in the treatment of aggressive lymphomas and has been related to relapse free survival (De Vita *et al.*, 1988). There is evidence suggesting a steep dose-response relationship for cisplatin (Drewinko *et al.*, 1973; Ozols *et al.*, 1984; Ozols *et al.*, 1985; Levin & Hryniuk, 1987). However a study in advanced germ cell tumour proved that doubling the dose of cisplatin (from $20\,mg\,m^{-2}$ for five consecutive days to $40\,mg\,m^{-2}$) did not improve the outcome (Nichols *et al.*, 1991). Cisplatin toxicity on the other hand increases with higher doses (Drewinko *et al.*, 1973; Roelofs *et al.*, 1984; Campbell *et al.*, 1983; Kelsen *et al.*, 1985; Reddel *et al.*, 1982) and with increasing cumulative dose (Roelofs *et al.*, 1984; Dominici *et al.*, 1989). The method of drug administration is also important (Drewinko *et al.*, 1973; Roelofs *et al.*, 1984; Posner *et al.*, 1986). Doses up to $200\,mg\,m^{-2}$ per course, either as a daily bolus for 5 days or by continuous infusion have been given with an important but acceptable increase in toxicity (Ozols *et al.*, 1985; Dominici *et al.*, 1989; Ozols *et al.*, 1988). We have used an intermediate cisplatin dose ($60\,mg\,m^{-2}$ per course). Other investigators have used $100\,mg\,m^{-2}$ per course, either as a continuous one day infusion (Velasquez *et al.*, 1988; Cabanillas *et al.*, 1988; Philip *et al.*, 1991; Press *et al.*, 1991) or a daily bolus for 4–5 days (Goss *et al.*, 1991; Nichols *et al.*, 1988). The response rates and survival in these trials are similar. It thus remains unproved that moderate increase in cisplatin dose intensity results in a greater response rate and survival in lymphoma.

Long term disease free survival in patients with resistant or relapsed aggressive lymphomas treated with conventional chemotherapy is extremely low. Efforts should probably be directed towards improving the results of first line treatments in patients with poor prognostic features. EPIC stands as a second line chemotherapy regimen with a good overall response rate and a low toxicity profile and could be a useful combination for cytoreduction prior to high dose chemotherapy. The search for a satisfactory regimen for resistant disease needs to be continued.

Dr Hickish is a CRC funded Senior Registrar and Dr Cunningham was a CRC funded Senior Lecturer.

### References

ACHTERRATH, W., NIEDERLE, N., RAETTIG, R. & HILGARD, P. (1982). Etoposide. Chemistry, preclinical and clinical pharmacology. *Cancer Treat. Rev.*, **9**, (Suppl A), 3–13.

ACKER, B., HOPPE, R.T., COLBY, T.V., COX, R.S., KAPLAN, H.S. & ROSEMBERG, S.A. (1983). Histologic conversion in the non-Hodgkin's lymphomas. *J. Clin. Oncol.*, **1**, 11–16.

APPELBAUM, F.R., SULLIVAN, K.M., BUCKNER, C.D., CLIFT, R.A., DEEG, J., FEFER, A. et al. (1987). Treatment of Malignant Lymphomas in 100 patients with chemotherapy, total body irradiation, and marrow transplantation. *J. Clin. Oncol.*, **5**, 1340–1347.

ARMITAGE, J.O., DICK, F.R. & CORDER, M.P. (1981). Diffuse histocytic lymphoma after histologic conversion: a poor prognostic variant. *Cancer Treat. Rep.*, **65**, 413–418.

CABANILLAS, F., RODRIGUEZ, V. & BODEY, G.P. (1980). Ifosfamide, methotrexate and vincristine (IMV) combination chemotherapy as secondary treatment for patients with malignant lymphoma. *Cancer Treat. Rep.*, **64**, 933–937.

CABANILLAS, F., HAGEMEISTER, F.B., BODEY, G.P. & FREIREICH, E.J. (1982). IMVP-16: an effective regimen for patients with lymphoma who have relapsed after initial combination chemotherapy. *Blood*, **60**, 693–697.

CABANILLAS, F., HAGEMEISTER, F.B., MCCLAUGHLIN, P., VELASQUEZ, W.S., RIGGS, S. et al. (1987). Results of MIME Salvage Regimen for Recurrent or Refractory Lymphoma. *J. Clin. Oncol.*, **5**, 407–412.

CABANILLAS, F., VELASQUEZ, W.S., MCLAUGHLIN, P., JAGANNATH, S., HAGEMEISTER, F.B., REDMAN, J.R. et al. (1988). Results of recent salvage chemotherapy regimens for lymphoma and Hodgkin's disease. *Semin. Hematol.*, **25**, (Suppl. 2), 47–50.

CABANILLAS, F., JAGANNATH, S. & PHILIP, T. (1990). Management of recurrent or refractory disease. In *The Non-Hodgkin's Lymphomas*, Magrath, I.T. (ed.) pp 359–372. Edward Arnold: London.

CAMPBELL, A.B., KALMAN, S.M. & JACOBS, C. (1983). Plasma platinum levels: relationship to cisplatin dose and nephrotoxicity. *Cancer Treat. Report*, **67**, 169–172.

CAVALLI, F., JUNGI, W.F., NISSEN, N.I., PAJAK, T.F., COLEMAN, M. & HOLLAND, J.F. (1981). Phase II trial of cis-dichlorodiammineplatinum (II) in advanced malignant lymphoma: a study of the cancer and acute leukemia group B. *Cancer*, **48**, 1927–1930.

CHABNER, B.A. & WILSON, W. (1991). Reversal of multidrug resistance. *J. Clin. Oncol.*, **9**, 4–6 (editorial).

COLVIN, M. (1982). The comparative pharmacology of cyclophosphamide and ifosfamide. *Semin. Oncol.*, **9**, (Suppl 1), 2–7.

COX, D.R. (1972). Regression models and life tables. *J.R. Stat. Soc., Series B*, **34**, 187–202.

DE VITA, V.T. Jr, HUBBARD, S.M., YOUNG, R.C. & LONGO, D.L. (1988). The role of chemotherapy in diffuse aggressive lymphoma. *Semin. Hematol.*, **25**, Suppl 2, 2–10.

DE VITA, V.T. Jr, JAFFE, E.S., MAUCH, P. & LONGO, D.L. (1989). Lymphocytic lymphomas. In *Cancer – Principles and Practice of Oncology*, De Vita, V.T. Jr., Hellman, S. & Rosenberg, S.A. (eds) pp 1741–1798. Lippincott: Philadelphia.

DOMINICI, C., PETRUCCI, F., CAROLI, S., ALIMONTI, A., CLERICO, A. & CASTELLO, M.A. (1989). A pharmacokinetic study of high-dose continuous infusion cisplatin in children with solid tumors. *J. Clin. Oncol.*, **7**, 100–107.

DREWINKO, B., BROWN, B.W. & GOTTLIEB, J.A. (1973). The effect of cis-diamminedichloroplatinum (II) on cultured human lymphoma cells and its therapeutic implications. *Cancer Res.*, **33**, 3091–3095.

DREWINKO, B., GREEN, C. & LOO, T.L. (1976). Combination chemotherapy *in vitro* with cis-dichlordiammine platinum (II). *Cancer Treat. Rep.*, **60**, 1619–1621.

DURAND, R.E. & GOLDIE, J.H. (1987). Interaction of etoposide and cisplatin in an *in vitro* tumor model. *Cancer Treat. Rep.*, **71**, 673–679.

FREI, III E., TEICHER, B.A., HOLDEN, S.A., CATHCART, K.N.S. & WANG, Y. (1988). Preclinical studies and clinical correlation of the effect if alkilating dose. *Cancer Res.*, **48**, 6417–6423.

GOLDIN, A. (1982). Ifosfamide in experimental tumor systems. *Semin. Oncol.*, **9** (Suppl. 1), 14–23.

GOSS, P.E., SHEPHERD, F.A., SCOTT, J.G., WARNER, E., BAKER, M.A., SUTTON, D. et al. (1991). Dexamethasone/ifosfamide/cisplatin/etoposide (DICE) as therapy for patients with advanced refractory non-Hodgkin's lymphoma: preliminary report of a phase II study. *Ann. Oncol.*, **2**, (Suppl. 1), 43–46.

HAGEMEISTER, F.B., TANNIR, N., MCLAUGHLIN, P., SALVADOR, P., RIGGS, S. et al. (1987). MIME Chemotherapy (Methyl-GAG, Ifosfamide, Methotrexate, Etoposide) as treatment for recurrent Hodgkin's disease. *J. Clin. Oncol.*, **5**, 556–561.

HILGARD, P., HERDRICH, K. & BRADE, W. (1983). Ifosfamide. Current aspects and perspectives. *Cancer Treat. Rev.*, **10**, (Suppl A), 183–192.

HUIJGENS, P.C., OSSENKOPPELE, G.J., VAN DER LELIE, J., THOMAS, L.L.M., WIJNGAARDEN, M.J. & REIJNEKE, R.M.R. (1988). Ifosfamide and VP-16213 combination chemotherapy combined with ablative chemotherapy and autologous marrow transplantation as salvage treatment for malignant lymphoma. *Eur. J. Cancer Clin. Oncol.*, **24**, 483–486.

JUDSON, I.R. & WILTSHAW, E. (1985). Cis-dichlorodiammine platinum (Cis-platinum) and etoposide (VP16) in malignant lymphoma - an effective salvage regimen. *Cancer Chemother. Pharmacol.*, **14**, 258-261.

KELSEN, D.P., ALCOCK, N. & YOUNG, C.W. (1985). Cisplatin nephrotoxicity. Correlation with plasma platinum concentrations. *Am. J. Clin. Oncol.*, **8**, 77-80.

LEHMANN, E.L. (1959). *Testing Statistical Hypotheses*. Wiley: New York.

LEVIN, L. & HRYNIUK, W.M. (1987). Dose intensity analysis of chemotherapy regimens in ovarian carcinoma. *J. Clin. Oncol.*, **5**, 756-767.

LONGO, D.L. (1990). The use of chemotherapy in the treatment of Hodgkin's disease. *Semin Oncol.*, **17**, 716-735.

MILLER, A.B., HOOGSTRATEN, B., STAQUET, M. & WINKLER, A. (1981). Reporting results of cancer treatment. *Cancer*, **47**, 207-214.

MILLER, T.P., GROGAN, T.M., DALTON, W.S., SPIER, C.M., SCHEPER, R.J. & SALMON, S.E. (1991). P-Glycoprotein expression in malignant lymphoma and reversal of clinical drug resistance with chemotherapy plus high-dose verapamil. *J. Clin. Oncol.*, **9**, 17-24.

NICHOLS, C.R., LOEHRER, P.J., GREIST, A., KUBILIS, P.S. & HOFFMAN, R. (1988). Salvage chemotherapy for lymphoma with VP-16, ifosfamide and cisplatin. *Med. Pediat. Oncol.*, **16**, 12-16.

NICHOLS, C.R., WILLIAMS, S.D., LOEHRER, P.J., GRECO, A., CRAWFORD, E.D., WEETLAUFER, J. et al. (1991). Randomized study of cisplatin dose intensity in poor-risk germ cell tumors: a southeastern cancer study group and southwest oncology group protocol. *J. Clin. Oncol.*, **9**, 1163-1172.

OZOLS, R.F., CORDEN, B.J., JACOB, J., WESLEY, M., OSTCHEGA, Y. & YOUNG, R.C. (1984). High dose cisplatin in hypertonic saline. *Ann. Intern. Med.*, **100**, 19-24.

OZOLS, R.F., OSTCHEGA, Y., MYERS, C.E. & YOUNG, R.C. (1985). High-dose cisplatin in hypertonic saline in refractory ovarian cancer. *J. Clin. Oncol.*, **3**, 1246-1250.

OZOLS, R.F., IHDE, D.C., LINEHAM, M. et al. (1988). A randomized trial of standard chemotherapy versus a high-dose chemotherapy regiment in the treatment of poor prognosis germ cell tumors. *J. Clin. Oncol.*, **6**, 1031-1040.

PETO, R., PIKE, M.C., ARMITAGE, P., BRESLOW, N.E., COX, D.R., HOWARD, S.V. et al. (1977). Design and analysis of randomized clinical trials requiring prolonged observation of each patient. II. Analysis and examples. *Br. J. Cancer*, **35**, 1-39.

PFREUNDSCHUH, M.G., SCHOPPE, W.D., FUCHS, R., PFLÜGER, K.H., LOEFFLER, M. & DIEHL, V. (1987). Lomustine, etoposide, vindesine and dexamethasone (CEVD) in Hodgkin's lymphoma refractory to cyclophosphamide, vincristine, procarbazine and prednisone (COPP) and doxorubicin, bleomycin, vinblastine and dacarbazine (ABVD): a multicenter trial of the German Hodgkin Study Group. *Cancer Treat. Rep.*, **71**, 1203-1207.

PHILIP, T., CHAUVIN, F., BRON, D., GUGLIELMI, C., HAGENBEEK, A., COIFFIER, B. et al. (1991). PARMA international protocol: pilot study on 50 patients and preliminary analysis of the ongoing randomized study (62 patients). *Ann. Oncol.*, **2**, (Suppl. 1), 57-64.

PHILIP, T., ARMITAGE, J.O., SPITZER, G., CHAUVIN, F., JAGANNATH, S. et al. (1987). High-dose chemotherapy and autologous bone marrow transplantation after failure of conventional chemotherapy in adults with intermediate-grade or high-grade non-Hodgkin's lymphoma. *N. Engl. J. Med.*, **316**, 1493-1498.

PHILLIPS, G.L., FAY, J.W., HERZIG, R.H., LAZARUS, H.M., WOLFF, S.N., LIN, H. et al. (1990). The treatment of progressive non-Hodgkin's lymphoma with intensive chemoradiotherapy and autologous marrow transplantation. *Blood*, **75**, 831-838.

PLOOY, A.C.M., VAN DIJK, M. & LOHMAN, P.H.M. (1984). Induction and repair of DNA cross-links in Chinese hamster ovary cells treated with various platinum coordination compounds in relation to platinum binding to DNA, cytotoxicity, mutagenicity and antitumour activity. *Cancer Res.*, **44**, 2043-2051.

POSNER, M.R., SKARIN, A.T., CLARK, J. & ERVIN, T.J. (1986). Phase I study of continuous infusion cisplatin. *Cancer Treat. Rep.*, **70**, 847-850.

PRESS, O.W., LIVINGSTON, R., MORTIMER, J., COLLINS, C. & APPELBAUM, F. (1991). Treatment of relapsed Non-Hodgkin's lymphomas with Dexamethasone, High dose Cytarabine and Cisplatin before marrow transplantation. *J. Clin. Oncol.*, **9**, 423-431.

REDDEL, R.R., KEFFORD, R.F., GRANT, J.M., COATES, A.S., FOX, R.M. & TATTERSALL, M.H.N. (1982). Ototoxicity in patients receiving cisplatin: importance of dose and method of drug administration. *Cancer Treat. Report*, **66**, 19-23.

RODRIGUEZ, V., MCCREDIE, K.B., KEATING, M.J., VALDIDIESO, M., BODEY, G.P. & FREIREICH, E.J. (1978). Isophosphamide therapy for hematologic malignancies in patients refractory to prior treatment. *Cancer Treat. Rep.*, **62**, 493-497.

ROELOFS, R.I., HRUSHESKY, W., ROGIN, J. & ROSENBERG, L. (1984). Peripheral sensory neuropathy and cisplatin chemotherapy. *Neurology*, (NY) **34**, 934-938.

SANTORO, A., VIVIANI, S., VALAGUSSA, P., BONFANTE, V. & BONADONNA, G. (1986). CCNU, Etoposide, and Prednimustine (CEP) in refractory Hodgkin's disease. *Semin. Oncol.*, **13**, (Suppl. 1), 23-26.

SCHABEL, F.M. Jr, TRADER, M.W., LASTER, W.R. Jr, CORBETT, T.H. & GRISWOLD, D.P. Jr (1979). Cisdichloro-diammine-platinum II: combination chemotherapy and cross-resistance studies with tumors of mice. *Cancer Treat. Rep.*, **63**, 1459-1473.

SCHEULEN, M.E., BREMER, K., NIEDERLE, N. & SEEBER, S. (1983). Treatment of refractory malignant lymphomas with ifosfamide/etoposide combination chemotherapy. *Cancer Treat. Rev.*, **10**, (Suppl. A), 137-143.

TAKVORIAN, T., CANELLOS, G.P., RITZ, J., FREEDMAN, A.S., ANDERSON, K.C. et al. (1987). Prolonged disease-free survival after autologous bone marrow transplantation in patients with non-Hodgkin's lymphomas with a poor prognosis. *N. Eng. J. Med.*, **316**, 1499-1505.

TAYLOR, R.E., MCELWAIN, T.J. & BARRETT, A. (1982). Etoposide as a single agent in relapsed advanced lymphomas. A phase II study. *Cancer Chemother. Pharmacol.*, **7**, 175-177.

TSENG, A. Jr, JACOBS, C., COLEMAN, C.N., HORNING, S.J., LEWIS, B.J. & ROSEMBERG, S.A. (1987). Third-line chemotherapy for resistant Hodgkin's disease with Lomustine, Etoposide and Methotrexate. *Cancer Treat. Rep.*, **71**, 475-478.

VELASQUEZ, W.S., CABANILLAS, F., SALVADOR, P. et al. (1988). Effective salvage therapy for lymphoma with cisplatin in combination with high-dose Ara-C and dexamethasone (DHAP). *Blood*, **71**, 117-122.

VOSE, J.M., BIERMAN, P.J. & ARMITAGE, J.O. (1990). Hogkin's disease: the role of bone marrow transplantation. *Semin. Oncol.*, **17**, 749-757.

ZWELLING, L.A. & KOHN, K.W. (1979). Mechanism of action of cis-dichlorodiammine platinum (II). *Cancer Treat. Rep.*, **63**, 1429-1444.


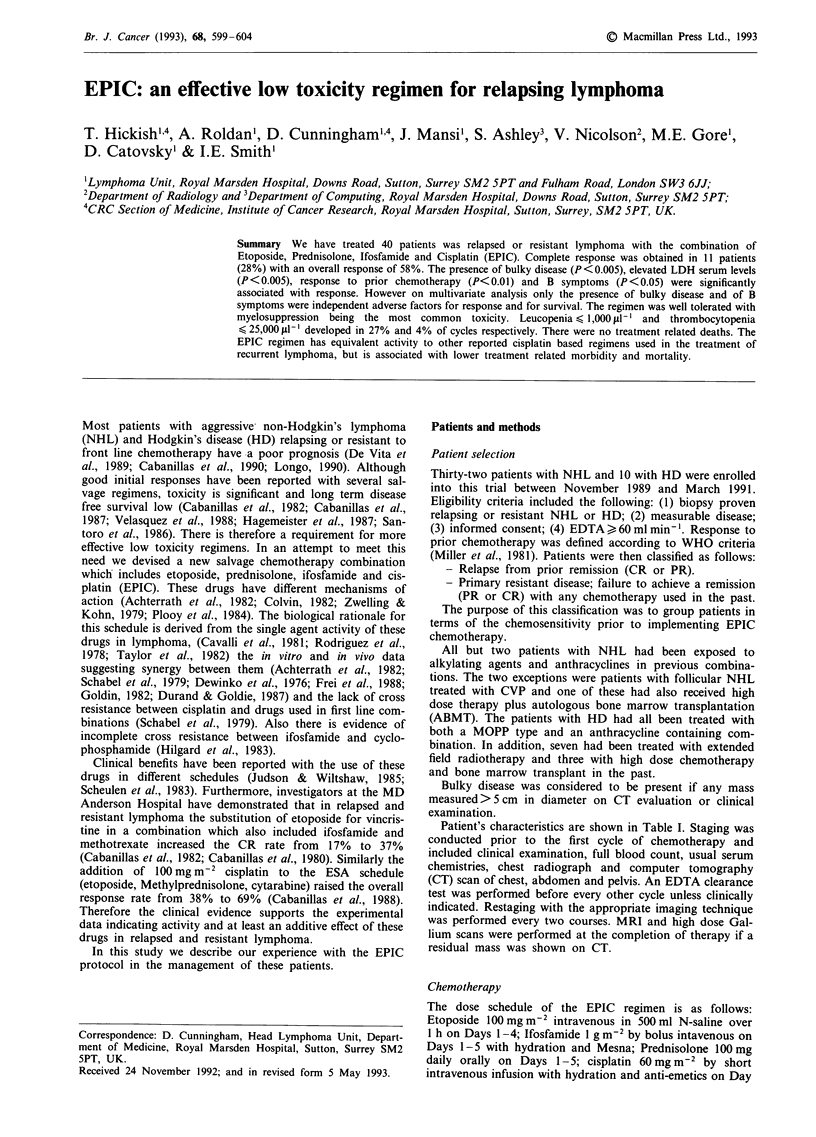

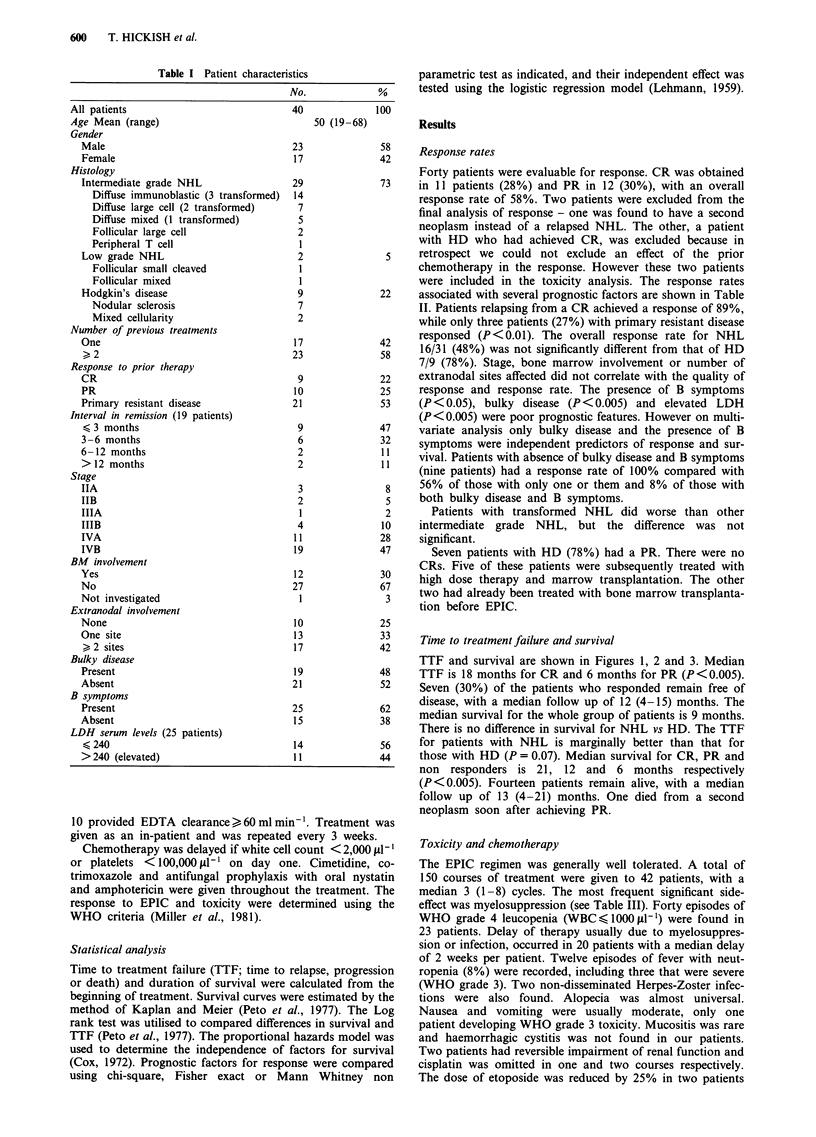

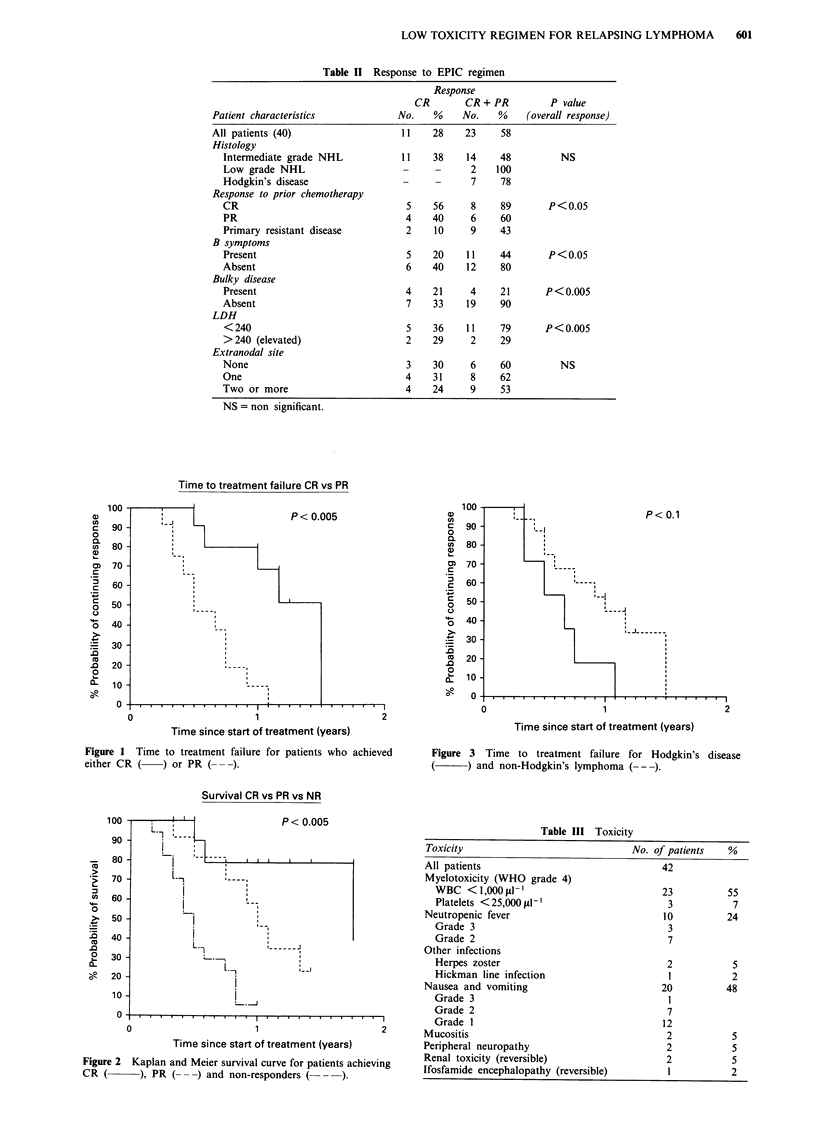

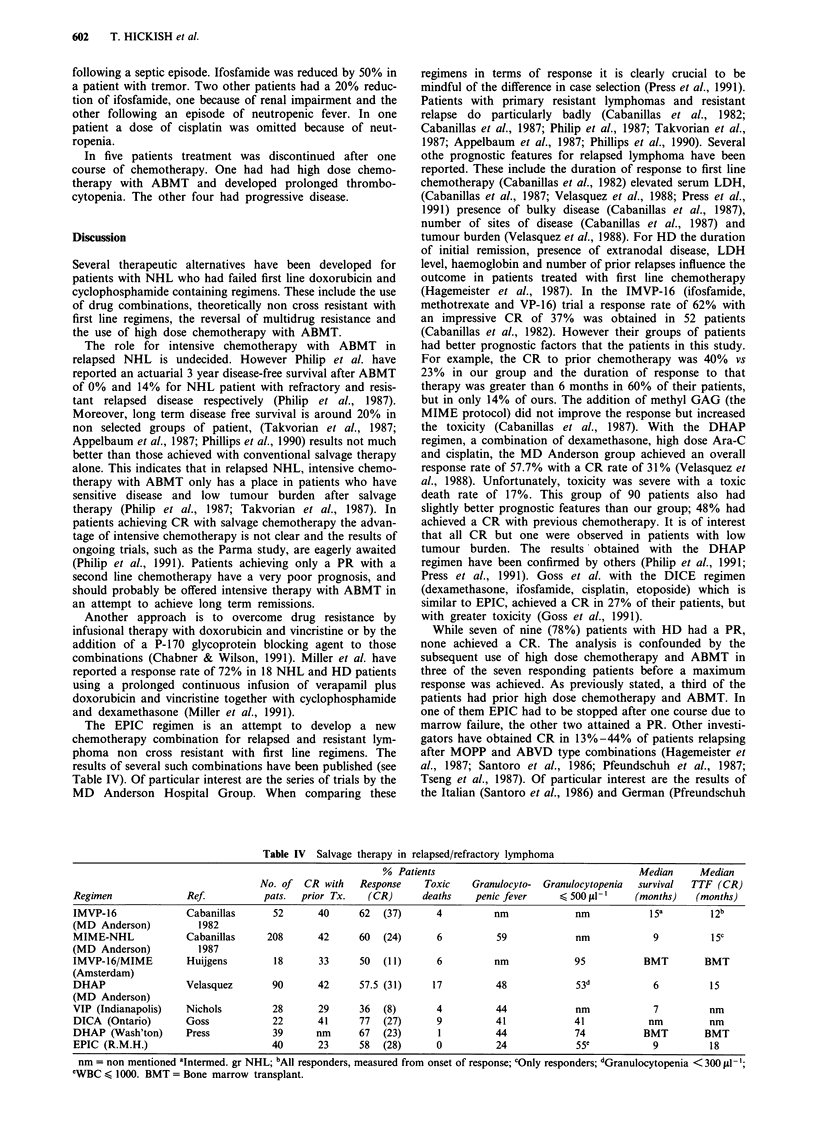

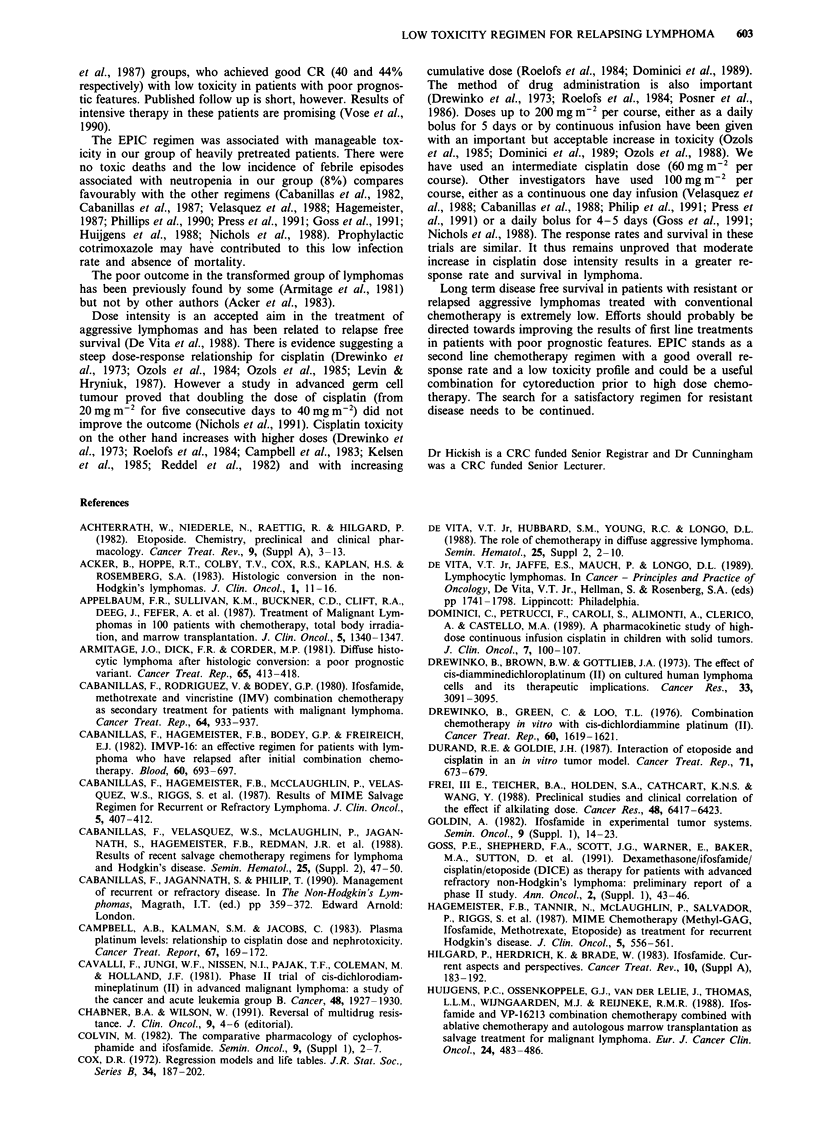

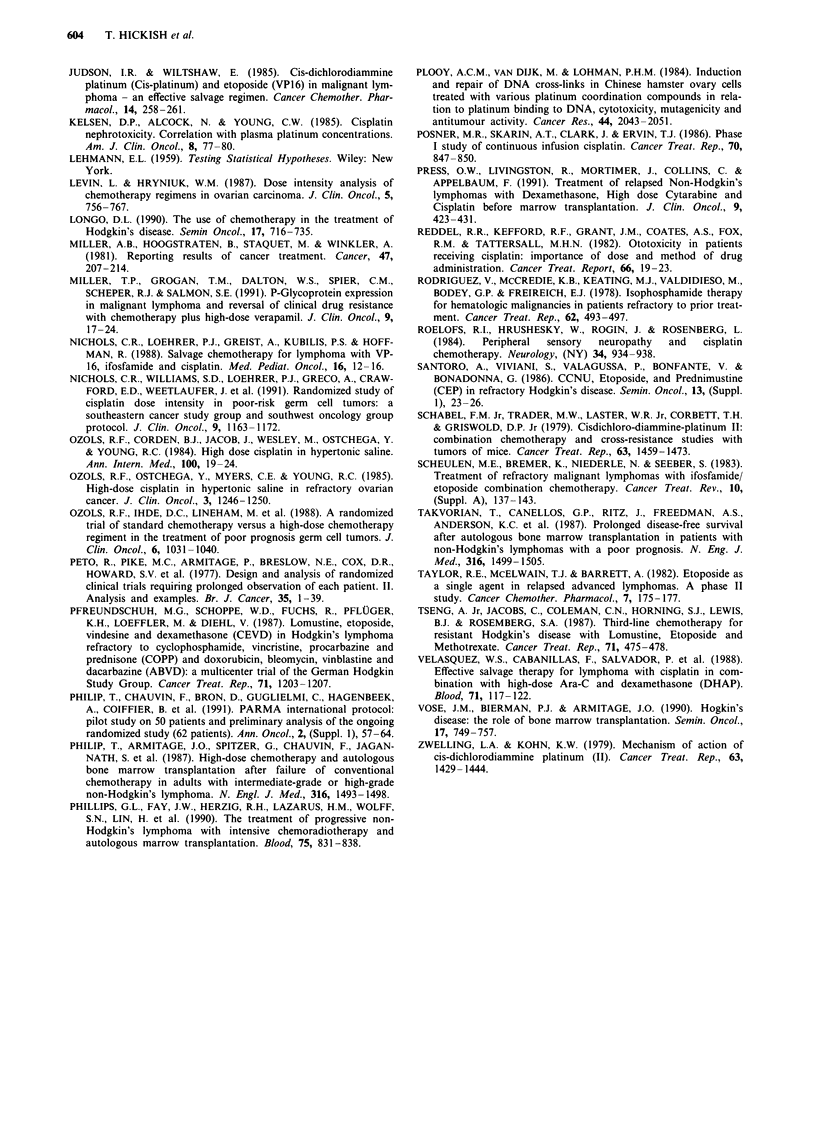


## References

[OCR_00833] Achterrath W., Niederle N., Raettig R., Hilgard P. (1982). Etoposide--chemistry, preclinical and clinical pharmacology.. Cancer Treat Rev.

[OCR_00838] Acker B., Hoppe R. T., Colby T. V., Cox R. S., Kaplan H. S., Rosenberg S. A. (1983). Histologic conversion in the non-Hodgkin's lymphomas.. J Clin Oncol.

[OCR_00843] Appelbaum F. R., Sullivan K. M., Buckner C. D., Clift R. A., Deeg H. J., Fefer A., Hill R., Mortimer J., Neiman P. E., Sanders J. E. (1987). Treatment of malignant lymphoma in 100 patients with chemotherapy, total body irradiation, and marrow transplantation.. J Clin Oncol.

[OCR_00848] Armitage J. O., Dick F. R., Corder M. P. (1981). Diffuse histiocytic lymphoma after histologic conversion: a poor prognostic variant.. Cancer Treat Rep.

[OCR_00859] Cabanillas F., Hagemeister F. B., Bodey G. P., Freireich E. J. (1982). IMVP-16: an effective regimen for patients with lymphoma who have relapsed after initial combination chemotherapy.. Blood.

[OCR_00867] Cabanillas F., Hagemeister F. B., McLaughlin P., Velasquez W. S., Riggs S., Fuller L., Smith T. (1987). Results of MIME salvage regimen for recurrent or refractory lymphoma.. J Clin Oncol.

[OCR_00853] Cabanillas F., Rodriguez V., Bodey G. P. (1980). Ifosfamide, methotrexate, and vincristine (IMV) combination chemotherapy as secondary treatment for patients with malignant lymphoma.. Cancer Treat Rep.

[OCR_00871] Cabanillas F., Velasquez W. S., McLaughlin P., Jagannath S., Hagemeister F. B., Redman J. R., Swan F., Rodriguez M. A. (1988). Results of recent salvage chemotherapy regimens for lymphoma and Hodgkin's disease.. Semin Hematol.

[OCR_00882] Campbell A. B., Kalman S. M., Jacobs C. (1983). Plasma platinum levels: relationship to cisplatin dose and nephrotoxicity.. Cancer Treat Rep.

[OCR_00887] Cavalli F., Jungi W. F., Nissen N. I., Pajak T. F., Coleman M., Holland J. F. (1981). Phase II trial of cis-dichlorodiammineplatinum (II) in advanced malignant lymphoma: a study of the cancer and acute leukemia group B.. Cancer.

[OCR_00904] DeVita V. T., Hubbard S. M., Young R. C., Longo D. L. (1988). The role of chemotherapy in diffuse aggressive lymphomas.. Semin Hematol.

[OCR_00915] Dominici C., Petrucci F., Caroli S., Alimonti A., Clerico A., Castello M. A. (1989). A pharmacokinetic study of high-dose continuous infusion cisplatin in children with solid tumors.. J Clin Oncol.

[OCR_00921] Drewinko B., Brown B. W., Gottlieb J. A. (1973). The effect of cis-diamminedichloroplatinum (II) on cultured human lymphoma cells and its therapeutic implications.. Cancer Res.

[OCR_00927] Drewinko B., Green C., Loo T. L. (1976). Combination chemotherapy in vitro with cis-dichlorodiammineplatinum(II).. Cancer Treat Rep.

[OCR_00932] Durand R. E., Goldie J. H. (1987). Interaction of etoposide and cisplatin in an in vitro tumor model.. Cancer Treat Rep.

[OCR_00937] Frei E., Teicher B. A., Holden S. A., Cathcart K. N., Wang Y. Y. (1988). Preclinical studies and clinical correlation of the effect of alkylating dose.. Cancer Res.

[OCR_00942] Goldin A. (1982). Ifosfamide in experimental tumor systems.. Semin Oncol.

[OCR_00946] Goss P. E., Shepherd F. A., Scott J. G., Warner E., Baker M. A., Sutton D., Farquharson H. A., Buick S., Sutcliffe S. (1991). Dexamethasone/ifosfamide/cisplatin/etoposide (DICE) as therapy for patients with advanced refractory non-Hodgkin's lymphoma: preliminary report of a phase II study.. Ann Oncol.

[OCR_00953] Hagemeister F. B., Tannir N., McLaughlin P., Salvador P., Riggs S., Velasquez W. S., Cabanillas F. (1987). MIME chemotherapy (methyl-GAG, ifosfamide, methotrexate, etoposide) as treatment for recurrent Hodgkin's disease.. J Clin Oncol.

[OCR_00959] Hilgard P., Herdrich K., Brade W. (1983). Ifosfamide--current aspects and perspectives.. Cancer Treat Rev.

[OCR_00964] Huijgens P. C., Ossenkoppele G. J., van der Lelie J., Thomas L. L., Wijngaarden M. J., Reijneke R. M. (1988). Ifosfamide and VP-16213 combination chemotherapy combined with ablative chemotherapy and autologous marrow transplantation as salvage treatment for malignant lymphoma.. Eur J Cancer Clin Oncol.

[OCR_00974] Judson I. R., Wiltshaw E. (1985). Cis-dichlorodiammineplatinum (cis-platinum) and etoposide (VP-16) in malignant lymphoma--an effective salvage regimen.. Cancer Chemother Pharmacol.

[OCR_00980] Kelsen D. P., Alcock N., Young C. W. (1985). Cisplatin nephrotoxicity. Correlation with plasma platinum concentrations.. Am J Clin Oncol.

[OCR_00989] Levin L., Hryniuk W. M. (1987). Dose intensity analysis of chemotherapy regimens in ovarian carcinoma.. J Clin Oncol.

[OCR_00994] Longo D. L. (1990). The use of chemotherapy in the treatment of Hodgkin's disease.. Semin Oncol.

[OCR_00998] Miller A. B., Hoogstraten B., Staquet M., Winkler A. (1981). Reporting results of cancer treatment.. Cancer.

[OCR_01003] Miller T. P., Grogan T. M., Dalton W. S., Spier C. M., Scheper R. J., Salmon S. E. (1991). P-glycoprotein expression in malignant lymphoma and reversal of clinical drug resistance with chemotherapy plus high-dose verapamil.. J Clin Oncol.

[OCR_01017] Nichols C. R., Williams S. D., Loehrer P. J., Greco F. A., Crawford E. D., Weetlaufer J., Miller M. E., Bartolucci A., Schacter L., Einhorn L. H. (1991). Randomized study of cisplatin dose intensity in poor-risk germ cell tumors: a Southeastern Cancer Study Group and Southwest Oncology Group protocol.. J Clin Oncol.

[OCR_01022] Ozols R. F., Corden B. J., Jacob J., Wesley M. N., Ostchega Y., Young R. C. (1984). High-dose cisplatin in hypertonic saline.. Ann Intern Med.

[OCR_01032] Ozols R. F., Ihde D. C., Linehan W. M., Jacob J., Ostchega Y., Young R. C. (1988). A randomized trial of standard chemotherapy v a high-dose chemotherapy regimen in the treatment of poor prognosis nonseminomatous germ-cell tumors.. J Clin Oncol.

[OCR_01027] Ozols R. F., Ostchega Y., Myers C. E., Young R. C. (1985). High-dose cisplatin in hypertonic saline in refractory ovarian cancer.. J Clin Oncol.

[OCR_01038] Peto R., Pike M. C., Armitage P., Breslow N. E., Cox D. R., Howard S. V., Mantel N., McPherson K., Peto J., Smith P. G. (1977). Design and analysis of randomized clinical trials requiring prolonged observation of each patient. II. analysis and examples.. Br J Cancer.

[OCR_01044] Pfreundschuh M. G., Schoppe W. D., Fuchs R., Pflüger K. H., Loeffler M., Diehl V. (1987). Lomustine, etoposide, vindesine, and dexamethasone (CEVD) in Hodgkin's lymphoma refractory to cyclophosphamide, vincristine, procarbazine, and prednisone (COPP) and doxorubicin, bleomycin, vinblastine, and dacarbazine (ABVD): a multicenter trial of the German Hodgkin Study Group.. Cancer Treat Rep.

[OCR_01060] Philip T., Armitage J. O., Spitzer G., Chauvin F., Jagannath S., Cahn J. Y., Colombat P., Goldstone A. H., Gorin N. C., Flesh M. (1987). High-dose therapy and autologous bone marrow transplantation after failure of conventional chemotherapy in adults with intermediate-grade or high-grade non-Hodgkin's lymphoma.. N Engl J Med.

[OCR_01053] Philip T., Chauvin F., Bron D., Guglielmi C., Hagenbeek A., Coiffier B., Gisselbrecht C., Kluin Nelemans J. C., Somers R., Misset J. C. (1991). PARMA international protocol: pilot study on 50 patients and preliminary analysis of the ongoing randomized study (62 patients).. Ann Oncol.

[OCR_01065] Phillips G. L., Fay J. W., Herzig R. H., Lazarus H. M., Wolff S. N., Lin H. S., Shina D. C., Glasgow G. P., Griffith R. C., Lamb C. W. (1990). The treatment of progressive non-Hodgkin's lymphoma with intensive chemoradiotherapy and autologous marrow transplantation.. Blood.

[OCR_01071] Plooy A. C., van Dijk M., Lohman P. H. (1984). Induction and repair of DNA cross-links in chinese hamster ovary cells treated with various platinum coordination compounds in relation to platinum binding to DNA, cytotoxicity, mutagenicity, and antitumor activity.. Cancer Res.

[OCR_01078] Posner M. R., Skarin A. T., Clark J., Ervin T. J. (1986). Phase I study of continuous-infusion cisplatin.. Cancer Treat Rep.

[OCR_01083] Press O. W., Livingston R., Mortimer J., Collins C., Appelbaum F. (1991). Treatment of relapsed non-Hodgkin's lymphomas with dexamethasone, high-dose cytarabine, and cisplatin before marrow transplantation.. J Clin Oncol.

[OCR_01090] Reddel R. R., Kefford R. F., Grant J. M., Coates A. S., Fox R. M., Tattersall M. H. (1982). Ototoxicity in patients receiving cisplatin: importance of dose and method of drug administration.. Cancer Treat Rep.

[OCR_01096] Rodriguez V., McCredie K. B., Keating M. J., Valdivieso M., Bodey G. P., Freireich E. J. (1978). Isophosphamide therapy for hematologic malignancies in patients refractory to prior treatment.. Cancer Treat Rep.

[OCR_01102] Roelofs R. I., Hrushesky W., Rogin J., Rosenberg L. (1984). Peripheral sensory neuropathy and cisplatin chemotherapy.. Neurology.

[OCR_01107] Santoro A., Viviani S., Valagussa P., Bonfante V., Bonadonna G. (1986). CCNU, etoposide, and prednimustine (CEP) in refractory Hodgkin's disease.. Semin Oncol.

[OCR_01113] Schabel F. M., Trader M. W., Laster W. R., Corbett T. H., Griswold D. P. (1979). cis-Dichlorodiammineplatinum(II): combination chemotherapy and cross-resistance studies with tumors of mice.. Cancer Treat Rep.

[OCR_01119] Scheulen M. E., Bremer K., Niederle N., Seeber S. (1983). Treatment of refractory malignant lymphomas with ifosfamide/etoposide combination chemotherapy.. Cancer Treat Rev.

[OCR_01125] Takvorian T., Canellos G. P., Ritz J., Freedman A. S., Anderson K. C., Mauch P., Tarbell N., Coral F., Daley H., Yeap B. (1987). Prolonged disease-free survival after autologous bone marrow transplantation in patients with non-Hodgkin's lymphoma with a poor prognosis.. N Engl J Med.

[OCR_01132] Taylor R. E., McElwain T. J., Barrett A., Peckham M. J. (1982). Etoposide as a single agent in relapsed advanced lymphomas. A phase II study.. Cancer Chemother Pharmacol.

[OCR_01137] Tseng A., Jacobs C., Coleman C. N., Horning S. J., Lewis B. J., Rosenberg S. A. (1987). Third-line chemotherapy for resistant Hodgkin's disease with lomustine, etoposide, and methotrexate.. Cancer Treat Rep.

[OCR_01143] Velasquez W. S., Cabanillas F., Salvador P., McLaughlin P., Fridrik M., Tucker S., Jagannath S., Hagemeister F. B., Redman J. R., Swan F. (1988). Effective salvage therapy for lymphoma with cisplatin in combination with high-dose Ara-C and dexamethasone (DHAP).. Blood.

[OCR_01149] Vose J. M., Bierman P. J., Armitage J. O. (1990). Hodgkin's disease: the role of bone marrow transplantation.. Semin Oncol.

[OCR_01154] Zwelling L. A., Kohn K. W. (1979). Mechanism of action of cis-dichlorodiammineplatinum(II).. Cancer Treat Rep.

